# Generation of tunable dual X-ray pulses in synchrotron light sources

**DOI:** 10.1107/S1600577525007374

**Published:** 2025-09-10

**Authors:** Jingye Xu, Haisheng Xu, Na Wang

**Affiliations:** a Institute of High Energy Physics Chinese Academy of Sciences Beijing China; b University of the Chinese Academy of Sciences,Beijing China; SESAME, Jordan

**Keywords:** double X-ray pulses, electron storage ring, synchrotron light source, two X-ray pulses, tunable separations, over-stretching, X-ray-pump/X-ray-probe

## Abstract

Synchrotron light sources are powerful platforms for cutting-edge, multidisciplinary research, with dozens currently in operation, construction or commissioning worldwide. It is widely recognized that different research areas have specific demands for source capabilities. For the majority of synchrotron facilities, delivering high-brightness, high-flux synchrotron radiation stably through high-current electron beams is the primary mode of operation. However, there is also a significant demand for timing experiments to study dynamic processes. In response, many synchrotron facilities offer specialized or hybrid timing modes. In this paper, we propose a novel method that combines a double RF system and deflecting cavities to generate pairs of X-ray pulses in electron storage rings with adjustable transverse separation and time delay. This approach holds the potential to enable new experimental possibilities, such as X-ray pump and X-ray probe studies, or capturing information from two temporal points in a dynamic process using two X-ray probes simultaneously. We analyze the fundamental characteristics of this method, present a conceptual design, and demonstrate through simulations that the scheme can achieve adjustable transverse separation of the two X-ray pulses with tunable time delay on the order of 100 ps.

## Abstract 2

A novel method employing a double RF system and transverse deflecting cavities is proposed to generate tunable double X-ray pulses with adjustable transverse separation and time delay in electron storage rings. This approach offers new experimental possibilities such as X-ray pump and X-ray probe studies.

## 1. Introduction

Synchrotron light sources are among the most powerful platforms for multi-disciplinary research, supporting cutting-edge investigations across a variety of fields. Currently, there are over 50 synchrotron light sources either operational or under construction worldwide (Zhao, 2018 ▸). Each source is capable of providing synchrotron light to dozens of beamlines simultaneously, playing a crucial role in fostering innovation and advancing scientific research. The development of fourth-generation synchrotron light sources, based on diffraction-limited storage rings (Borland *et al.*, 2014 ▸; Eriksson *et al.*, 2014 ▸; Hettel, 2014 ▸; Tavares *et al.*, 2014 ▸; Liu *et al.*, 2014 ▸; Streun *et al.*, 2018 ▸; Jiao *et al.*, 2018 ▸; Xu *et al.*, 2023 ▸; Xu *et al.*, 2025 ▸; Raimondi *et al.*, 2023 ▸), has significantly increased brightness—several orders of magnitude higher than that of typical third-generation sources.

The extensive diversity in users’ research fields generates various demands on synchrotron facilities. To meet these diverse needs, numerous operational modes have been developed and implemented. The most commonly used scheme, known as the ‘multi-bunch mode’, involves storing electron beams with a high average current by filling the ring with many bunches of small to medium single-bunch charges, providing highly stable and bright synchrotron light. A variant, ‘the camshaft mode’ (also known as ‘hybrid mode’) (Sun *et al.*, 2012 ▸), satisfies primary requirements for high brightness and stability while accommodating time-resolved experiments by integrating ‘camshaft’ bunches with high single-bunch charges into the gaps between standard bunch trains.

Studying the dynamic evolution of phenomena like micro-structures is a key scientific objective for synchrotron facilities. Consequently, operation modes facilitating such studies are in high demand and have been implemented using various techniques. These include laser slicing (Zholents & Zolotorev, 1996 ▸; Schoenlein *et al.*, 2000 ▸; Di Mitri *et al.*, 2019 ▸), tuning the lattice into a low momentum compaction mode (Martin *et al.*, 2011 ▸; Tordeux *et al.*, 2012 ▸), using high-charge bunches (Wulff *et al.*, 1997 ▸; Emery *et al.*, 2009 ▸; Jiao *et al.*, 2018 ▸; Xu *et al.*, 2023 ▸), filling particles into transverse resonance island buckets (Ries *et al.*, 2015 ▸; Holldack *et al.*, 2022 ▸) and generating short bunches with transverse deflecting cavities (TDCs, also known as ‘crab cavities’) (Zholents *et al.*, 1999 ▸; Borland, 2005 ▸; Huang, 2016 ▸; Huang *et al.*, 2019 ▸; Huang *et al.*, 2023 ▸). Among these techniques, the work by Holldack *et al.* (2022 ▸) has demonstrated a method involving the storage of electron beams in transverse resonance island buckets, resulting in spatial separation in the *y*-direction. This approach allows manipulated X-ray beams to converge at the same sample point after passing through tailored beamline optics, showcasing the potential of utilizing spatial separation for enhanced experimental configurations. These diverse modes reflect the adaptability and commitment of synchrotron light sources to support a broad range of scientific inquiries.

To further expand the applications of synchrotron light sources and enhance opportunities for user experiments, building on these insights, including inspirations from Holldack’s work on *y*-direction separation, we propose a novel operation scheme that generates two X-ray pulses with both transverse separation and tunable time delay, adjustable from hundreds of picoseconds to nanoseconds. Longitudinal separation is achieved through higher harmonic cavities (HHCs) under overstretching conditions, while transverse separation is realized using TDCs. By adjusting the HHC and TDC settings, both longitudinal and transverse separations can be modified. Notably, transverse separation can be generated locally and subsequently recovered by precisely tuning the positions and settings of the upstream and downstream TDCs, ensuring other beamlines remain unaffected. In our studies, the *elegant* code (Borland, 2000 ▸) was utilized for particle tracking simulations, and the *SPECTRA* code (Tanaka & Kitamura, 2001 ▸; Tanaka, 2021 ▸) was used to simulate the generated synchrotron radiation.

This scheme offers new opportunities for users interested in studying dynamic processes. Potential applications include X-ray pump and X-ray probe experiments and single-shot probing of different sample positions. The ability to finely control spatial and temporal separations can greatly enhance the versatility and applicability of synchrotron light sources in advanced research.

The rest of the paper is organized as follows. Section 2 provides a detailed description of the proposed method, including the principles of the HHCs and TDCs. In Section 3, we explore studies on the influence of multipole magnets ‘feed-down’ effects in the insertion section between two TDCs. In Section 4, we present an example using the storage ring lattice of the High Energy Photon Source in its preliminary design to demonstrate the effectiveness and tunable nature of the two X-ray pulses. Finally, conclusions and discussions are provided in Section 5.

## 2. Generating tunable dual X-ray pulses using HHCs and TDCs

**Figure 1:**
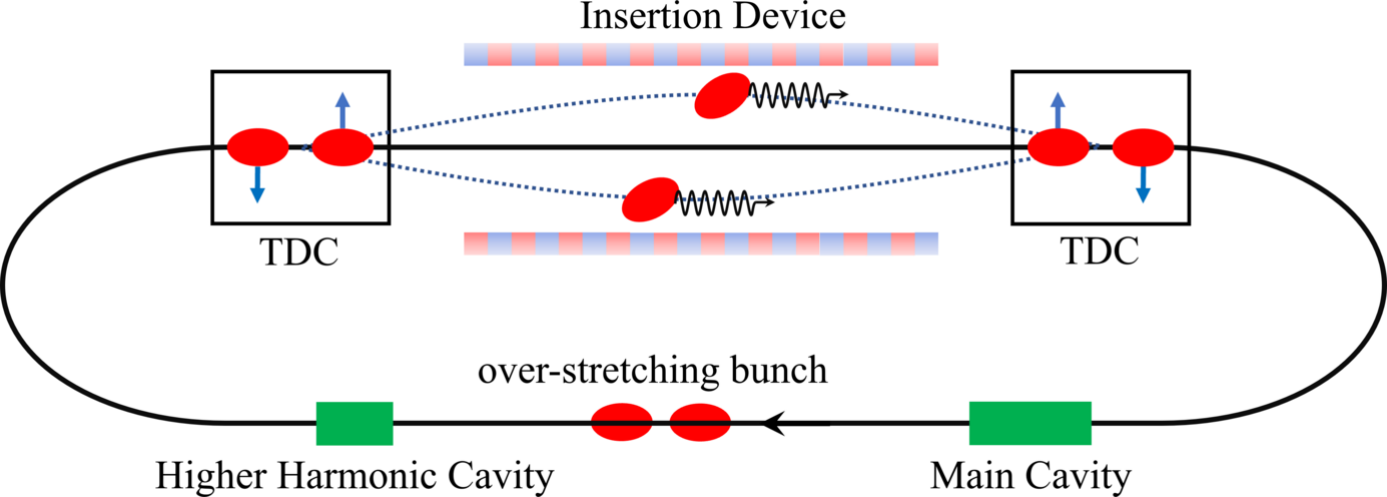
Schematic drawing of the proposed method for generating tunable dual X-ray pulses in an electron storage ring of a synchrotron light source. The green blocks represent the main cavity and HHC, which are used to create the overstretching condition and generate two microbunches with adjustable longitudinal separation. The TDCs are employed to achieve transverse separation of the microbunches within the insertion device while ensuring that the transverse separation remains transparent to other users.

To generate two X-ray pulses with tunable separations both longitudinally and transversely, we propose the simultaneous implementation of HHCs and TDCs. Fig. 1 ▸ illustrates the basic layout and principle of the proposed method. The core idea is to utilize a double RF system, comprising HHCs and main cavities, to significantly lengthen the electron bunch to overstretching conditions. By adjusting the double RF system’s settings, the electron bunch can be split into two microbunches longitudinally, with a tunable separation between them. Concurrently, two sets of TDCs are employed to modulate and reverse modulate the beam transversely, allowing the two microbunches to be separated transversely in the insertion devices in between the two TDCs. The transverse separation can be adjusted by tuning the TDCs settings. The use of two sets of TDCs ensures that the transverse separation remains localized, preventing any impact on the rest of the ring and keeping the scheme transparent to other users.

**Figure 2:**
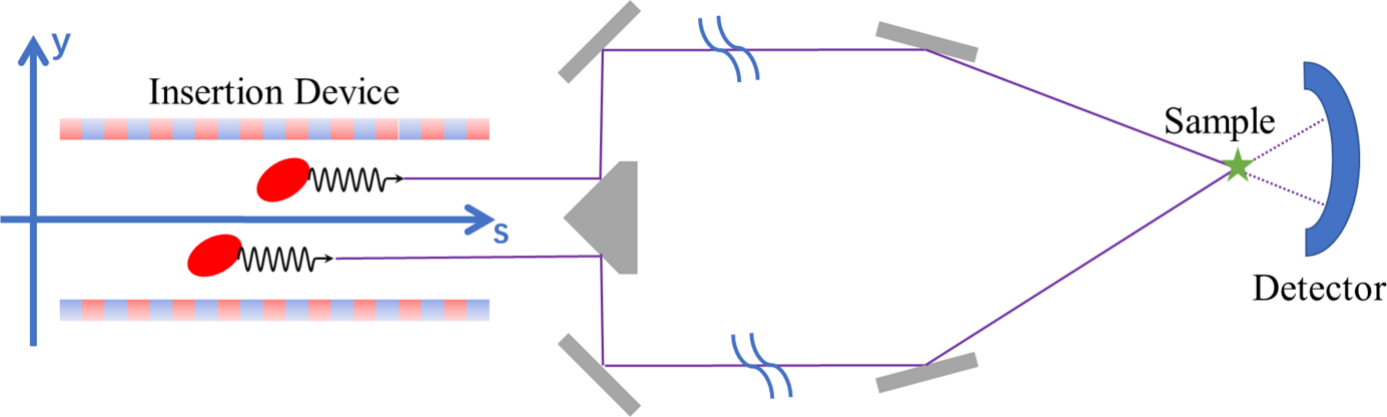
Schematic of the beamline design concept of two vertically separated X-ray pulses.

The *y*-displaced two electron microbunches result in two X-ray pulses with vertical separation. This spatial separation ensures that the trajectories of the two X-ray pulses are distinct, enabling their independent manipulation within the beamline. Such manipulations include monochromatization, focusing and delay adjustment, which collectively provide enhanced experimental flexibility, as depicted schematically in Fig. 2 ▸.

In this configuration, the vertically separated microbunches generate two X-ray pulses with distinct initial vertical positions. As these pulses propagate along the beamline, they are split into separate trajectories. After traversing designated sections of the beamline, the two X-ray pulses converge at the same sample location and are subsequently separated again at the detector. This setup allows processing to occur at a common interaction point, aligning with the typical requirement of pumping and probing the sample at the same location. The distinct trajectories, however, permit the differentiation of sample information carried by each pulse via strategically positioned detectors.

HHCs are widely utilized in synchrotron light sources, especially in fourth-generation facilities, primarily to lengthen electron bunches. By properly configuring the main cavities and HHCs, the shape of the RF buckets can be modified. This allows for straightforward division of an electron bunch into two ‘microbunches’ along the longitudinal axis by setting the main RF cavities and HHCs under ‘overstretching conditions’. The longitudinal time separation between the microbunches can be tuned by varying the RF voltages.

To achieve longitudinal separation of a bunch, one can start by utilizing the synchrotron equations of motion in a double RF system [see, for instance, equations (3.13) and (3.191) of Lee (2018 ▸)] to derive a longitudinal Hamiltonian for a double-RF system in the longitudinal phase space (ϕ, δ), 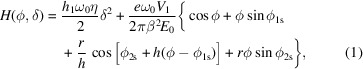
where ϕ_1s_ and ϕ_2s_ are the synchronous phases of the main cavity and the HHC, respectively. The term *h* = *h*_2_/*h*_1_ represents the ratio of the harmonic numbers of the HHCs and the main cavities, where *h*_1_ and *h*_2_ are the harmonic numbers for the main cavities and HHCs, respectively. Here, ω_0_ denotes the angular revolution frequency, and η = α_c_ − 1/γ^2^ is the phase slippage factor, with α_c_ the momentum compaction factor and γ the Lorentz factor. Furthermore, *r* = *V*_2_/*V*_1_ is the ratio of the peak voltages of the HHCs (*V*_2_) and the main cavities (*V*_1_), and *E*_0_ is the energy of the reference particles.

Starting from the fact that the phase space distribution is a function of the Hamiltonian [Ψ(ϕ, δ) = Ψ(*H*)] (Chao, 1993 ▸; Venturini, 2018 ▸), and considering that the energy distribution is Gaussian, we can derive the longitudinal distribution ρ(ϕ),
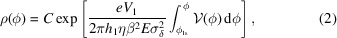
where *C* is the normalization constant, and 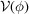 is 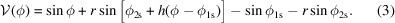
By increasing the voltage ratio *r*, through a rise in the peak voltage of the HHCs, the bunch length tends to increase accordingly. When *r* is set appropriately, an ideally flat potential is achieved, known as the ‘ideal lengthening condition’ or ‘optimal lengthening condition’. Further increasing *r* fulfills the overstretching conditions, enabling realization and adjustment of the longitudinal separation between the two microbunches.

While the two microbunches are separated longitudinally, distinguishing and manipulating the synchrotron light they generate is still challenging, or, at least, not flexible enough. One difficulty is that they propagate exactly in the same trajectory, with their close proximity typically on the order of 100 ps. Therefore, achieving transverse separation is crucial to enhance the flexibility and the practical applications of this scheme. The use of TDCs to provide different transverse kicks to the microbunches is a logical approach, as their longitudinal separation means they encounter the TDCs at different phases, indicating that the experienced kick by the two microbunches is different.

The transverse kick provided by a TDC, Δ*p*_⊥_(*z*), is given by 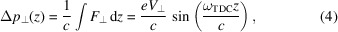
where *z* = −*C*_ring_(ϕ − ϕ_1s_)/(2π*h*_1_) is the longitudinal distance between a particle and the reference particle, *V*_⊥_ is the peak voltage of the TDCs, *c* is the speed of light in a vacuum, *F*_⊥_ is the vertical kick force given by the TDC, and ω_TDC_ is the angular frequency of the TDC. Through this equation [equation (4)], it can be observed that the transverse kicks are opposite in sign for *z* > 0 and *z* < 0. Aligning the microbunches around peaks with a 180° phase difference efficiently achieves transverse separation without requiring excessively high TDC voltages.

The design of TDCs involves careful determination of key parameters, such as frequency and peak voltage. For identical deflection of bunches in arbitrary buckets, the TDC frequency should be an integer multiple of the main RF frequency.

**Figure 3:**
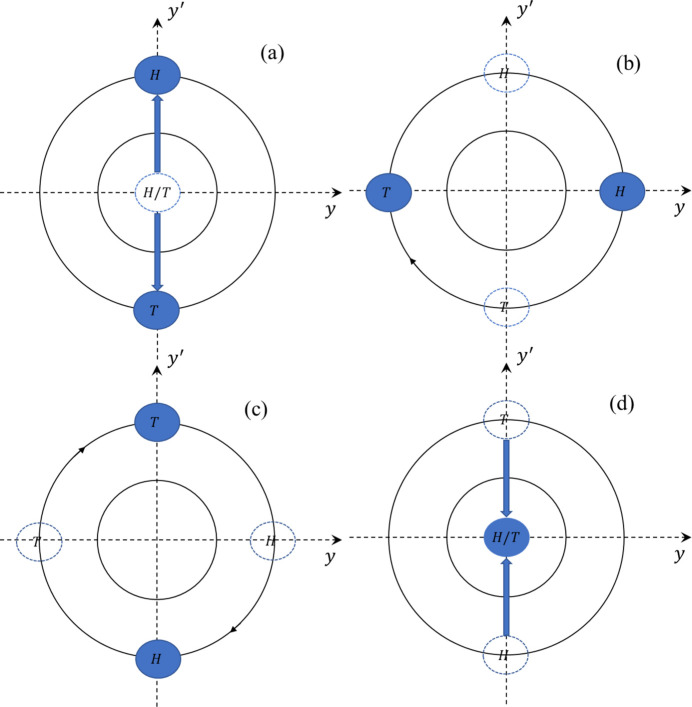
Illustration of the transverse separation process for two microbunches using TDCs. (*a*) At TDC1, the microbunches, due to their longitudinal separation, receive equal but opposite transverse kicks, maximizing their separation while maintaining control over transverse oscillations. (*b*) After a 90° betatron phase advance, the microbunches achieve their maximum transverse separation, which is ideal for insertion device placement to generate dual X-ray pulses with both longitudinal and transverse separation. (*c*) After another 90° phase advance, the microbunches reach TDC2. (*d*) At TDC2, opposite transverse kicks are applied, returning the microbunches to the center of the transverse phase space, ensuring no impact on other beamline operations.

By placing two sets of TDCs upstream and downstream of the insertion devices, with a betatron phase difference of 180° between them, the transverse separation can be confined locally. This ensures that the microbunches’ transverse separation does not affect other regions of the ring, indicating that the other beamlines are not going to be affected. Fig. 3 ▸ illustrates the evolution of the two microbunches in the transverse phase space. When the overstretched bunch arrives at TDC1, the longitudinal separation of the two microbunches results in them encountering different phases at TDC1. To optimize the separation within the insertion device (ID) and prevent excessive transverse oscillation for either microbunch, TDC1 imparts equal but opposite transverse kicks, as shown in Fig. 3 ▸(*a*). Following this, the microbunches undergo a 90° betatron phase advance, reaching the state depicted in Fig. 3 ▸(*b*), where they achieve maximum transverse separation. This is the optimal position for placing the insertion device, allowing the beamline to benefit from both transverse and longitudinal separation, producing dual X-ray pulses. To ensure that this separation does not affect other beamline users, a second TDC is strategically placed at the location where the microbunches have completed another 90° betatron phase advance, as shown in Fig. 3 ▸(*c*). This second TDC provides kicks with the same magnitude but opposite direction, allowing the microbunches to return to the center of the transverse phase space, as illustrated in Fig. 3 ▸(*d*). This setup ensures that the transverse separation is effectively localized to the ID, safeguarding the operation for other users.

## 3. Analyses of the ‘feed-down’ effects of the multipole magnets

In the previous section we illustrated the basic principles of our proposal. It is important to consider that the vertical separation of the two microbunches between the two sets of TDCs causes the particles to travel off-axis vertically through magnets in this region. This off-axis trajectory induces ‘feed-down’ effects from multipole magnets such as quadrupole, sextupole and octupole magnets. However, the impact of these ‘feed-down’ effects on particle motion varies significantly among different types of multipole magnets, necessitating a detailed, type specific analysis as follows.

**Figure 4:**
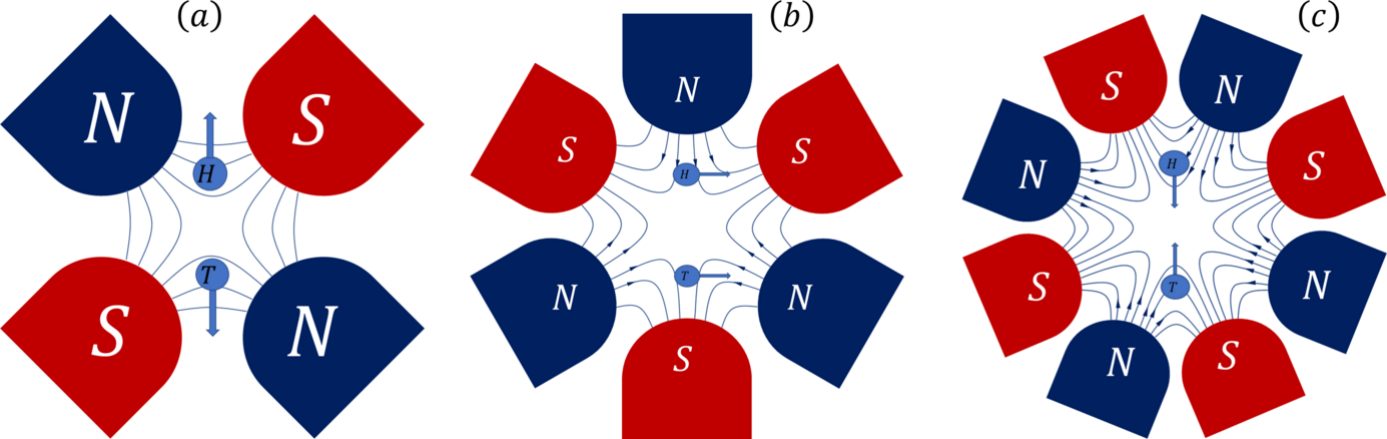
Schematic representation of the magnetic field lines and the forces experienced by vertically separated microbunches as they pass through different types of magnets. (*a*) In a quadrupole magnet, the upper microbunch is subjected to an upward force, while the lower microbunch experiences a downward force, affecting their vertical separation. (*b*) In a sextupole magnet, both microbunches experience forces in the same horizontal direction, potentially leading to horizontal offsets or coupling effects. (*c*) In an octupole magnet, the forces resemble those in a quadrupole, similarly affecting beam dynamics.

To understand how the feed-down effect influences particle motion, we analyzed the magnetic field distribution and the resulting forces experienced by the particles. Fig. 4 ▸ presents a schematic of the magnetic field lines and corresponding force directions. In Fig. 4 ▸(*a*), a sketch of a quadrupole magnet is shown. As the two vertically separated electron microbunches move perpendicularly out of the page through the quadrupole magnet, the upper microbunch encounters an upward magnetic force, while the lower microbunch experiences a downward force. This modulates the amplitude of their vertical separation as they transit the quadrupole, a phenomenon that can, in principle, be compensated for by optimizing the TDC settings.

Conversely, when the vertically separated microbunches transit a sextupole magnet, both upper and lower microbunches experience a magnetic force in the same horizontal direction, as shown in Fig. 4 ▸(*b*). This presents a more challenging effect, potentially causing a horizontal offset in the entire bunch through coupling.

A schematic of the magnetic field lines of an octupole magnet is illustrated in Fig. 4 ▸(*c*). The vertically separated microbunches experience forces that push them either toward or away from the magnet’s center, similar to the effect observed in a quadrupole. However, it is important to note that the field in an octupole is nonlinear, and the ‘feed-down’ effects of an octupole differ significantly from those in a quadrupole. In contrast, sextupoles predominantly influence the beam in the horizontal direction, as shown in Fig. 4 ▸(*b*).

The previous analyses were primarily qualitative, based on physical intuition. We now proceed to analyze the magnetic flux density as given by (Lee, 2018 ▸)
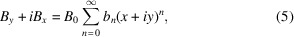
where *B*_0_ is the normalization constant, typically chosen as the main dipole field strength such that *b*_0_ = 1, *i.e.B*_0_*b*_0_ = [*B*ρ]/ρ, where [*B*ρ] is the momentum rigidity of the beam and ρ is the bending radius. *b*_*n*_ is the 2(*n* + 1)th normal multipole coefficients, defined as
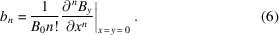
Assuming the vertical offset Δ*y* of a microbunch is significantly larger than the vertical closed orbit of the whole bunch *y*_0_ (Δ*y* ≫ *y*_0_), the changes in magnetic flux density due to feed-down effects in quadrupole, sextupole and octupole fields are expressed as
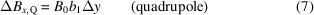

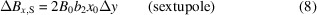

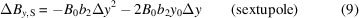

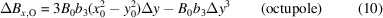

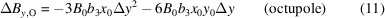
where Δ*B*_*x*,Q_ represents the extra horizontal magnetic field experienced by the particles passing through the quadrupole magnets with vertical offset. Δ*B*_*x*,S_ and Δ*B*_*y*,S_ are the horizontal and vertical magnetic field experienced by the particles passing through the sextupole magnets with vertical offset, respectively. Δ*B*_*x*,O_ and Δ*B*_*y*,O_ are the horizontal and vertical magnetic field experienced by the particles passing through the octupole magnets with vertical offset, respectively. *x*_0_ and *y*_0_ represent the horizontal and vertical closed orbits of the whole bunch, respectively.

From equation (7), when particles pass through a quadrupole with a vertical offset Δ*y*, an additional horizontal magnetic field proportional to Δ*y* arises. This horizontal field induces a vertical deflection force on the particles, known as the quadrupole feed-down effect (Pfingstner *et al.*, 2014 ▸). This effect can alter the beam’s vertical orbit. An important aspect of the quadrupole feed-down effect is that the direction of the horizontal field lines is determined by the sign of Δ*y*. Thus, when two microbunches pass symmetrically through quadrupoles with positive and negative vertical offsets, respectively, they experience opposing vertical deflection forces. However, this effect does not impact the betatron phase advance and, as such, should not lead to vertical orbit ‘leakage’, especially considering our plan to position the TDCs 180° apart in betatron phase advance.

Equations (8) and (9) reveal that passing through a sextupole with a vertical offset generates additional horizontal and vertical fields. The horizontal magnetic field in equation (8) is proportional to Δ*y* and the overall horizontal closed orbit. This means that, by correcting the beam’s overall horizontal closed orbit to near zero (*x*_0_ ≃ 0), the horizontal feed-down effects on the microbunches can be effectively minimized. Equation (9) shows that the additional vertical field is a summation of two components, proportional to Δ*y*^2^ and to *y*_0_Δ*y*. By reducing the beam’s overall vertical closed orbit (*y*_0_ ≃ 0), the second term can be effectively managed. The first term, proportional to Δ*y*^2^, suggests that, for microbunches with opposite vertical offsets, the induced horizontal deflection forces are in the same direction. This synchronized deflection can lead to horizontal orbit leakage outside the two TDCs.

In octupoles, as seen in equations (10) and (11), particles with a vertical offset may experience additional horizontal and vertical magnetic fields. In equation (10), the horizontal field has two components: one proportional to 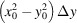 and another to Δ*y*^3^. The first component can be minimized by correcting the overall horizontal and vertical closed orbits. The second term, though expected to be numerically small, causes opposite horizontal deflections on the microbunches, affecting the vertical trajectory. Equation (11) indicates that the induced vertical field is directly linked to the overall path of the beam. By adjusting both the horizontal and vertical closed orbits, the impact of this feed-down effect can be substantially reduced.

From the theoretical analysis of feed-down effects in multipole magnets, it is evident that, within our proposal, the impact of sextupole feed-down effects requires careful examination. This is due to the horizontal forces experienced by particles passing vertically off-axis through sextupoles, necessitating thorough investigation to ensure local confinement of their effects.

## 4. Proof-of-principle case study

The High Energy Photon Source (HEPS) is the first fourth-generation synchrotron light source in China. To validate the proposed method, we utilized the HEPS storage ring lattice from its preliminary design stage as a ‘toy’ lattice. The primary parameters of this lattice are presented in Table 1 ▸. It is worth mentioning that the frequency selected for the main RF system of the HEPS storage ring is 166.6 MHz, indicating the maximum bucket length of approximately 6 ns. Additionally, active third-harmonic cavities are employed for bunch lengthening. The use of active harmonic cavities provides greater flexibility in shaping the longitudinal RF potential well to achieve overstretching conditions, allowing us to adjust the distance between the two microbunches effectively.

**Figure 5:**
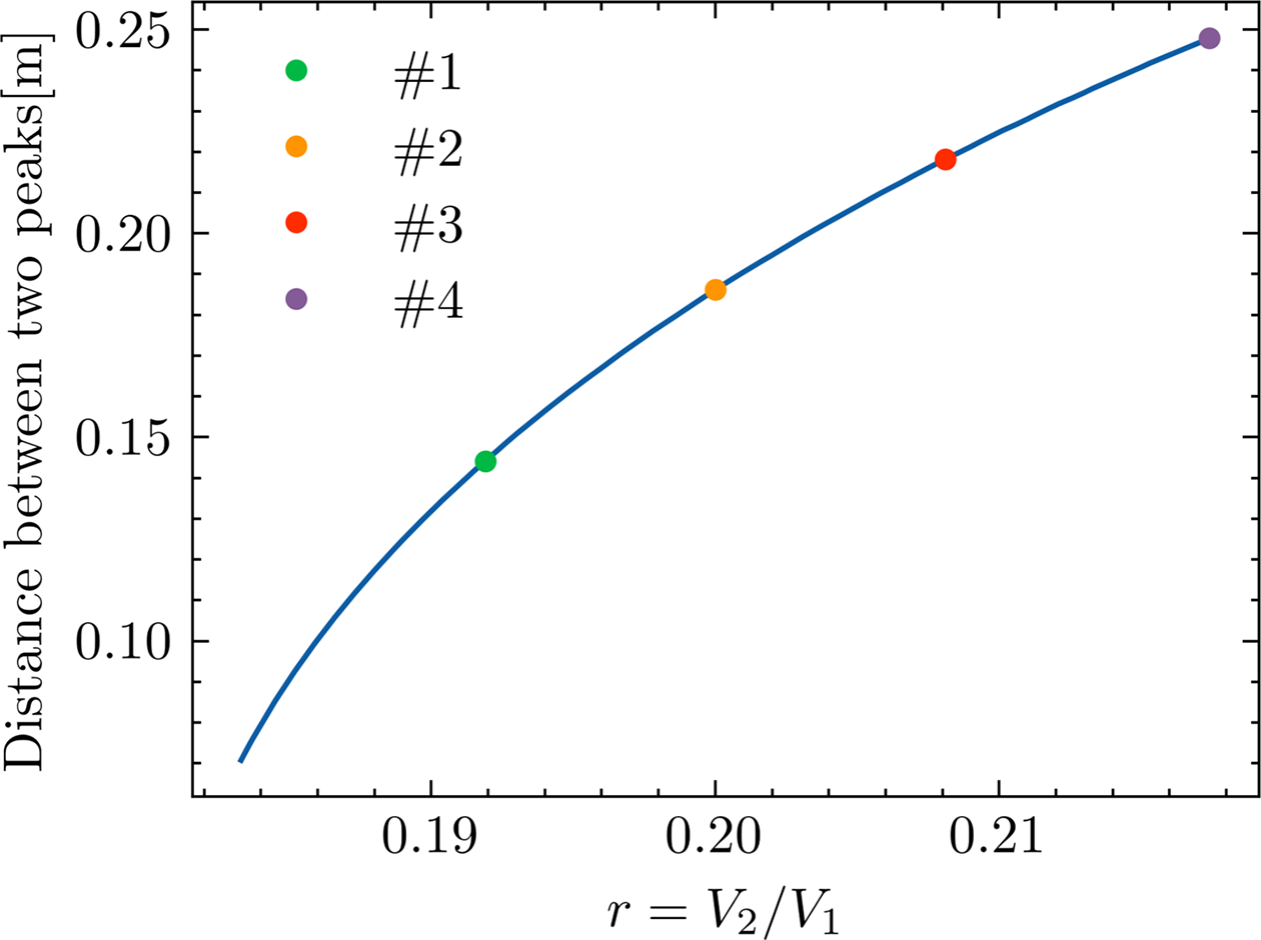
The relationship between the distance between two microbunches (vertical axis) and the ratio *r* of the peak voltage between the harmonic cavities and the main RF cavities (horizontal axis) is shown. The dark blue solid line represents theoretical results. By adjusting *r*, a wide range of separations can be achieved. Four representative settings, labeled as Cases #1 to #4, are highlighted on the curve for further detailed case studies.

In the HEPS storage ring, we planned to implement a double RF system under the ‘ideal lengthening’ condition, where the ratio *r* of the peak voltages between the third harmonic cavities and the main RF cavities is approximately 0.18. From this setup, we adjusted *r* by changing *V*_2_ while keeping *V*_1_ constant, allowing us to modify the time interval between the two microbunches. Fig. 5 ▸ illustrates how the microbunch distance correlates with the ratio *r*, highlighting the tunability of their longitudinal separations via adjustments in the double RF system. We selected four typical settings of the harmonic cavities (HHCs), labeled as Cases #1 to #4, as examples. These settings exhibit longitudinal separations ranging from approximately 0.144 m to 0.248 m, corresponding to roughly 480 ps to 830 ps. It is important to note that the 6D tracking simulations were carried out using the *elegant* code to obtain the results presented below. Neither synchrotron radiation damping nor quantum excitation were included in these simulations.

**Figure 6:**
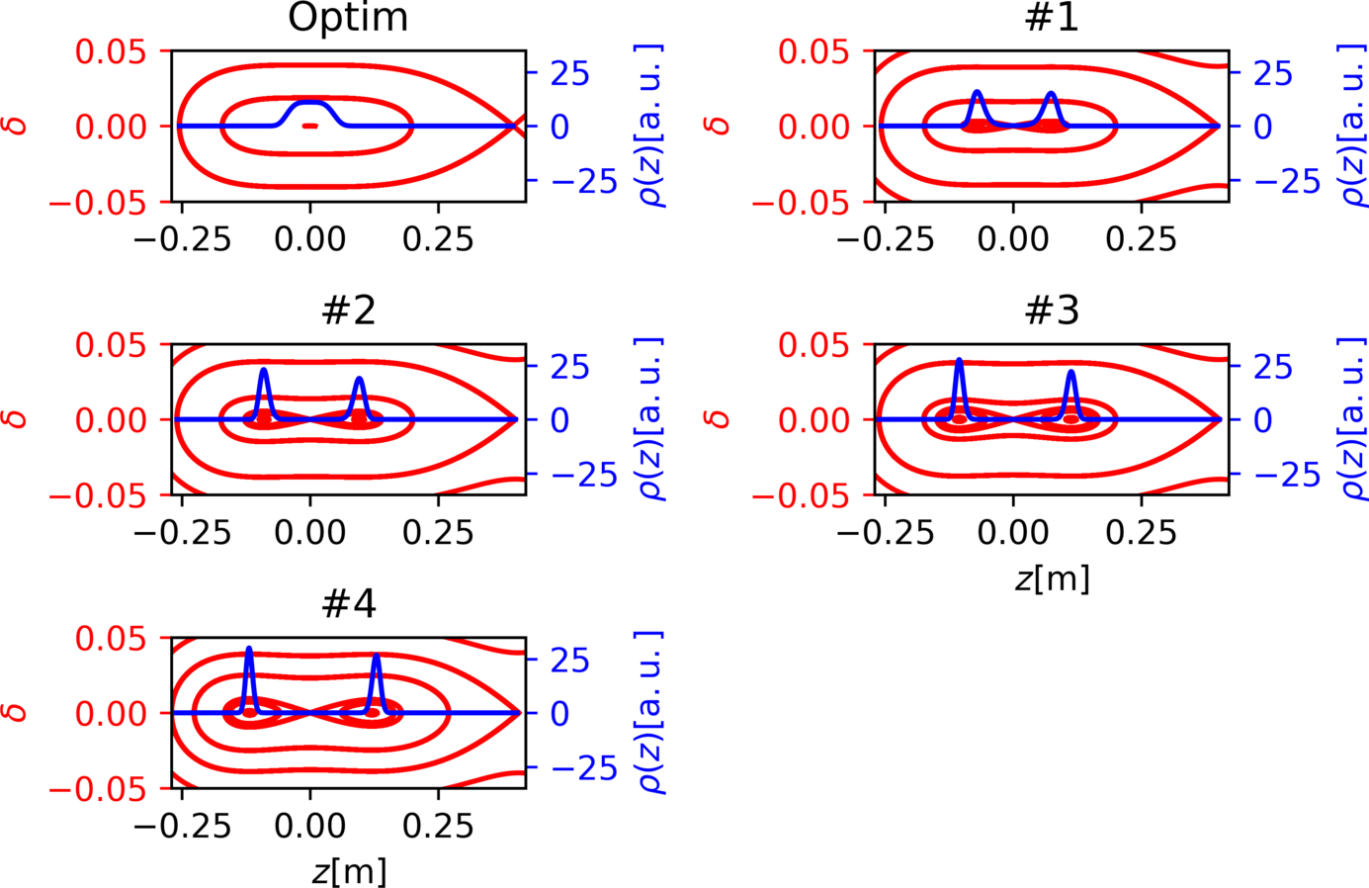
Hamiltonian torus (red) and normalized longitudinal distribution (blue) with five different settings. The first one, labeled ‘Optim’, shows the Hamiltonian tori and bunch longitudinal distribution under the ‘ideal lengthening’ condition of the double RF system. The other four subplots represent Case #1 to Case #4 in Fig. 5 ▸.

The longitudinal Hamiltonian tori and the corresponding bunch longitudinal distributions for the four cases labeled as Case #1 to Case #4 in Fig. 5 ▸ are displayed in Fig. 6 ▸. For ease of comparison, all subplots in Fig. 6 ▸ use the same scale for both the horizontal and vertical axes. Additionally, the first subplot in Fig. 6 ▸, labeled ‘Optim’, shows the Hamiltonian tori and bunch longitudinal distribution under the ‘ideal lengthening’ condition of the double RF system. It should be noted that these longitudinal distributions shown in Fig. 6 ▸, as well as all the computations presented in this paper, were simulated in the ‘zero-current limit’ and therefore do not incorporate any longitudinal impedance effects (Xu *et al.*, 2021 ▸; Xu *et al.*, 2024 ▸), which could result in microbunch lengthening if considered.

**Figure 7:**
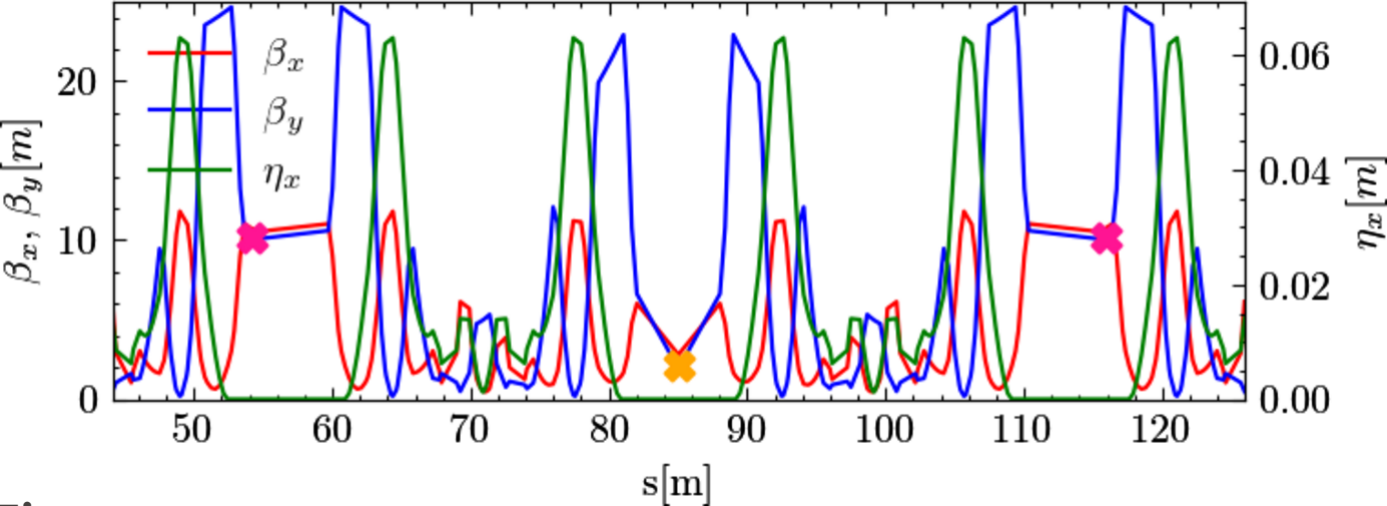
Twiss parameters for part of the ‘toy’ lattice. The red and blue curves represent the horizontal and vertical beta functions, respectively. The green curve is the horizontal dispersion function. The orange cross mark indicates the position of the light source point. The two red cross marks indicate the respective locations of the two TDCs.

Having discussed the double RF system settings, the corresponding longitudinal Hamiltonian tori, the variation trends in microbunch separation, and the longitudinal distribution for four typical settings, we now need to identify suitable locations for the TDCs in our ‘toy’ lattice. Ideally, the two TDCs should be placed 180° apart in betatron phase and 90° from the light source point. In practice, besides betatron phase differences, spatial layout must be considered to ensure adequate space at locations near a 90° phase difference from the light source point. Since we are considering vertical separation of the two microbunches, it is the vertical betatron phase difference that matters. Given that the insertion device is placed in a low-beta straight section of the ‘toy’ lattice, we set the light source point at the midpoint of this section, as indicated by the orange cross mark in Fig. 7 ▸.

Our toy lattice consists of 48 7BA cells. According to the vertical working point in Table 1 ▸, each 7BA cell has a vertical phase advance of approximately 4.43π. By placing TDCs in straight sections one 7BA cell away from the light source point and fine-tuning their position, we can achieve a precise vertical phase shift of 4.5π between the TDCs and the light source point. Fig. 7 ▸ shows the Twiss parameters for part of the ‘toy’ lattice. At intervals of one 7BA cell upstream and downstream of the light source point (orange cross mark), positions with a vertical betatron phase shift of 4.5π can be found for placing the two TDCs, indicated by red cross marks in Fig. 7 ▸.

The resonant frequency of the TDCs needs to be carefully determined. To ensure that all bunches in arbitrary buckets have identical separations, we have chosen the resonant frequency of the TDCs to be an integer harmonic of the main RF cavities. Specifically, we selected a harmonic number of four, meaning the TDC’s resonant frequency is four times the resonant frequency of the main RF, resulting in 666.4 MHz. Additionally, the availability of mature technology for the power source is an important factor in selecting the TDCs’ resonant frequency.

**Figure 8:**
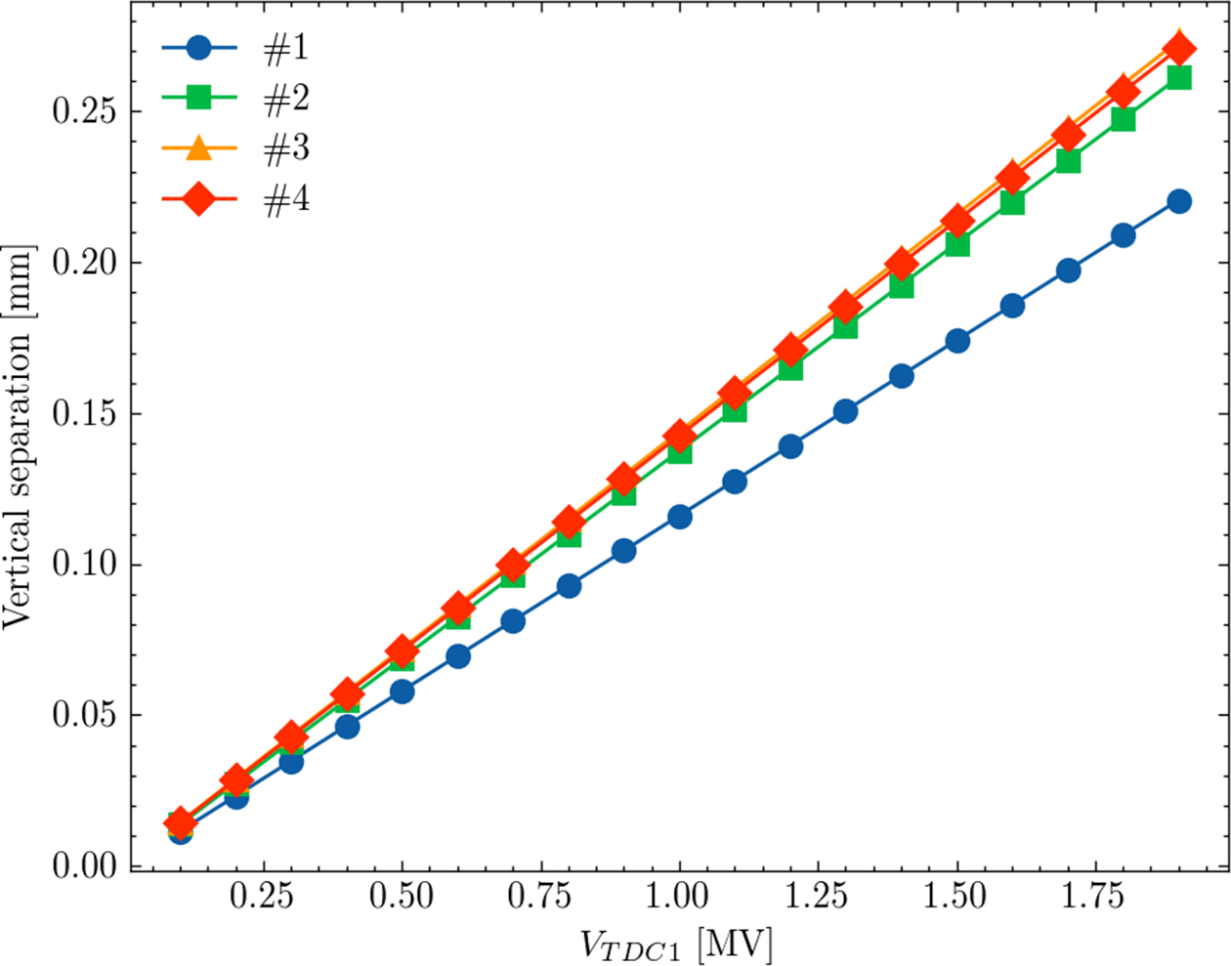
The vertical separation at the light source point when varying voltages of the TDC1.

In determining the voltage and phase settings for the deflecting cavity TDC1 located upstream of the light source point, we consider both the vertical separation and phase symmetry requirements. The voltage of TDC1 (*V*_TDC1_) is selected based on its proportional relationship with the transverse separation, as illustrated in Fig. 8 ▸. In our scenario, we opted for *V*_TDC1_ values that result in an approximate transverse separation of 100 µm. However, this voltage can be adjusted depending on specific user requirements.

In the *elegant* simulation, a deflecting cavity inverts the field direction at a phase of 90°. By default, this deflecting phase of 90° is referenced to the synchronous phase ϕ_1s_. For most over-stretching bunches, the phase of the two stable-fixed points (ϕ_SFP1_ and ϕ_SFP2_) are not symmetric about the synchronous phase ϕ_1s_. Therefore, to achieve symmetrical kicks, the phase of the first deflecting cavity ϕ_TDC1_ should be set to ϕ_TDC1_ = 0.5(ϕ_SFP1_ + ϕ_SFP2_) − ϕ_1s_ + 90°. This approach ensures that TDC1 operates effectively in maintaining bunch symmetry and separation.

**Figure 9:**
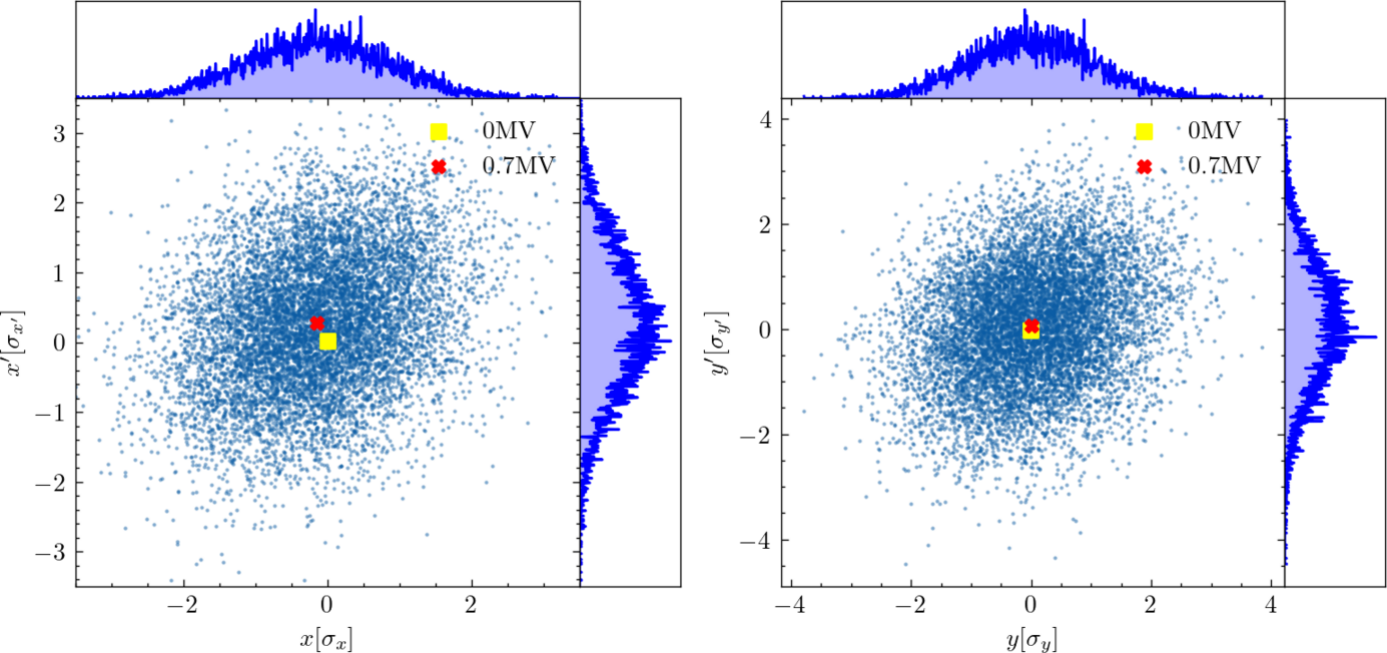
The left and right plots depict the macroparticles at the exit of the downstream deflecting cavity (TDC2) in the horizontal and vertical phase spaces, respectively. The yellow square marks the centroid coordinates of the bunch without the proposed scheme, while the red cross represents the centroid with the scheme applied.

To localize the vertical separation between the two microbunches, ensuring that the bunch parameters at the exit of the downstream deflecting cavity (TDC2) remain unchanged, we employed the differential evolution (DE) algorithm (Feoktistov, 2006 ▸) to optimize the parameters *V*_TDC2_ and ϕ_TDC2_ of TDC2. Following this optimization, the bunch distributions in both the horizontal and vertical phase spaces at the exit of TDC2 are depicted in the left and right plots of Fig. 9 ▸, respectively.

The yellow square and red cross markers in Fig. 9 ▸ indicate the centroid coordinates of the bunch at the exit of TDC2 without and with the proposed scheme applied, respectively. Despite the visual proximity of the markers, quantitative analysis reveals significant differences. Specifically, the normalized separations along the *x* and *x*′ axes are 15.2%σ_*xo*_ and 25.2%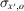, respectively, based on RMS sizes σ_*x*,*o*_ = 1.84 × 10^−5^ m and 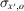 = 1.77 × 10^−6^. Additionally, the separations for *y* and *y*′ show 0.9% and 8.3% of their respective RMS values, which confirms that the observed separations are non-negligible.

The right-hand plot in Fig. 9 ▸ effectively shows the restoration of both the vertical centroid *y* and the vertical divergence *y*′. However, our prior analyses predicted, and the left-hand plot confirms, that displacement occurs in the horizontal phase space (*x*, *x*′), primarily due to vertical beam offsets at the sextupole magnets. Consequently, a comprehensive optimization of TDC2 is crucial to address the disturbances affecting the beam in the horizontal direction.

To mitigate the horizontal displacement observed at the exit of TDC2, we plan to re-optimize the field strength (*K*_2_) of the sextupole magnets situated between the two deflecting cavities. The optimization focuses on the following objectives:

(i) Position the two microbunches at the center of the horizontal phase space at the exit of TDC2;

(ii) Preserve the first-order chromaticities;

(iii) Minimize alterations to the *K*_2_ values of the sextupoles located between the two TDCs.

We employed the DE algorithm to optimize the centroids of the microbunches in the horizontal phase space at the exit of TDC2. This optimization aimed to reduce the offset from the center while attempting to maintain the first-order chromaticity by adjusting the strength of the sextupoles (*K*_2_) located between the two TDCs. In implementing the DE algorithm, we allowed each sextupole’s field strength to vary independently within a specified range of 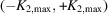, where 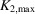 is the maximum absolute sextupole strength used in the toy lattice. The optimization successfully restored the centroid offset in the horizontal phase space at the exit of TDC2 to near-center, with changes in all sextupole field strengths kept within ±20% of their nominal values. Subsequently, by optimizing the voltage and phase of TDC2, we were able to bring the vertical phase space at the exit of TDC2 back to its intended state. As a result, for Case #1 through Case #4, we obtained optimized TDCs’ voltage, phase and frequency settings, along with a new configuration for the sextupoles between the two TDCs. The specific details are provided in Table 2 ▸.

**Figure 10:**
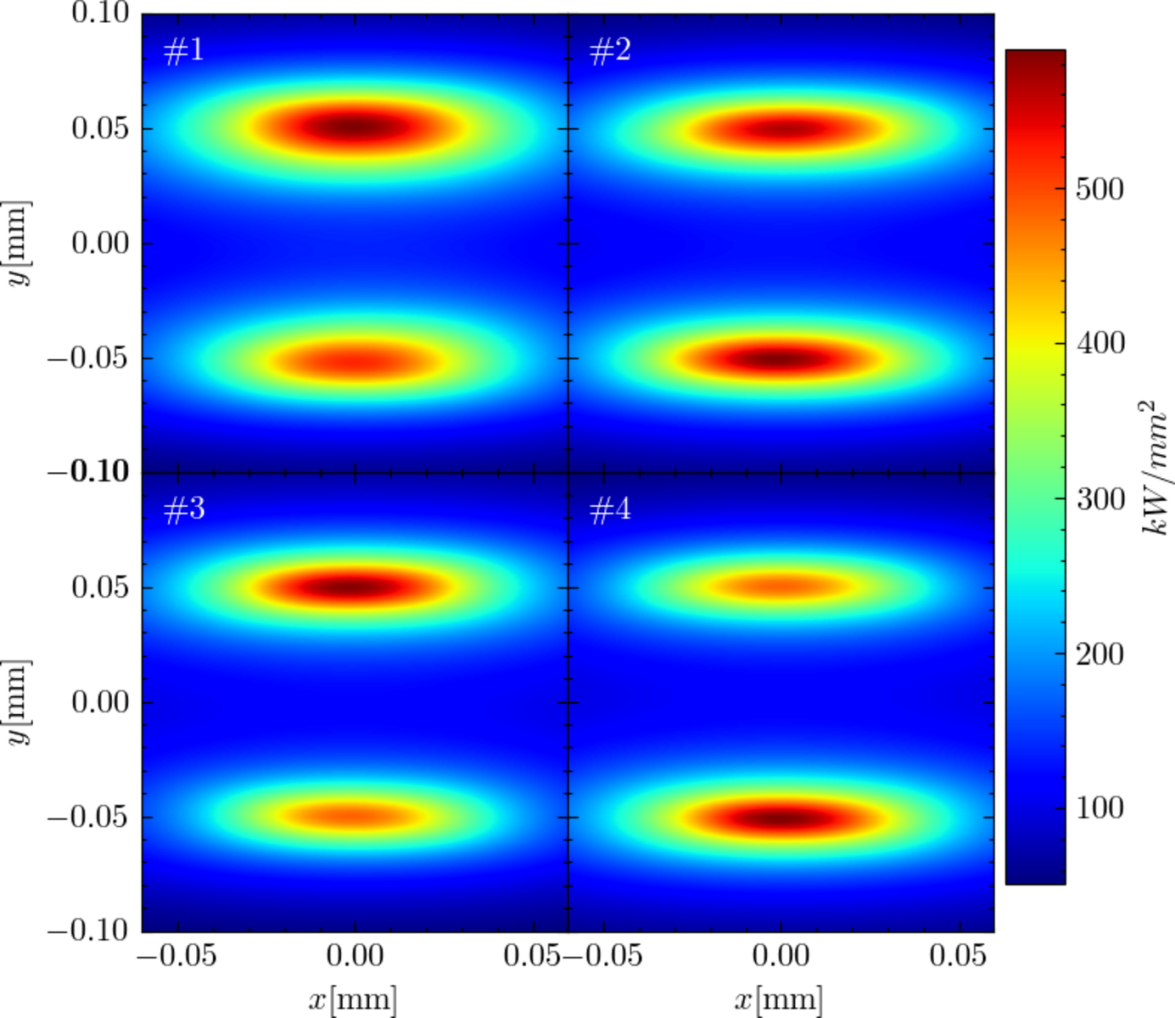
Power density of the vertically separated double X-ray pulses corresponding to the aforementioned four cases (from Case #1 to Case #4).

Using the settings obtained from the optimization of the four cases, we conducted simulations of the generated X-ray parameters with the *SPECTRA* code. In addition to the bunch distributions at the light source point derived from the lattices of the four cases, the key beam parameters and insertion device specifications used in the simulations are detailed in Table 3 ▸. Fig. 10 ▸ displays the transverse distribution of the light spot located 1.1 m downstream from the light source point, where redder colors indicate higher power density. It is evident from the results for all four cases that stable generation of vertically separated double X-ray pulses has been achieved. These simulation results, derived for four cases of different time delays by independently adjusting the TDC parameters, yielded four configurations with nearly consistent transverse separation. We believe that these findings provide strong evidence of the flexibility of our proposed idea, demonstrating its capability to produce double X-ray pulses with adjustable transverse separation and longitudinal time delay.

Moreover, Fig. 10 ▸ reveals slight differences in X-ray beam spot sizes across the four cases, with Case #1 exhibiting a slightly larger spot size than the others. These variations in vertical size growth are attributed to the different slopes of the TDC RF waveform encountered by the microbunches, which affect the *y*–*z* tilt. Although the figure shows variations in light spot sizes, these differences remain relatively modest. Furthermore, adjustments to spot size can be effectively managed through post-processing techniques, such as using pinholes on the beamline or employing transverse focusing of the generated X-rays.

**Figure 11:**
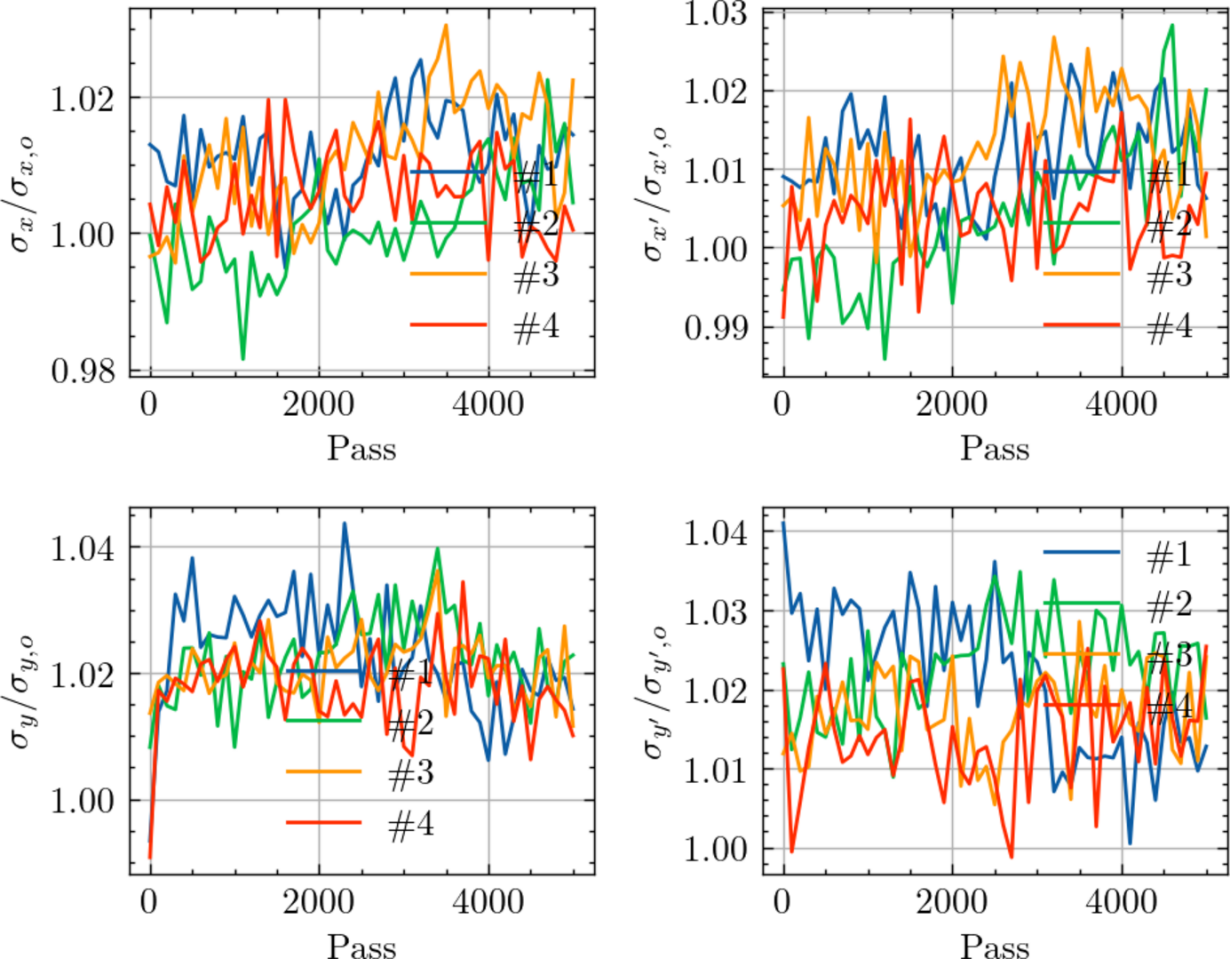
Comparative analysis of beam parameters growth at the midpoint of the adjacent downstream straight section of the TDC2, showing variations with and without TDCs across four cases. The top left and bottom left plots display the evolution of horizontal and vertical beam sizes (σ_*x*_ and σ_*y*_), respectively, relative to their ideal values (σ_*x*,*o*_ and σ_*y*,*o*_) over the number of turns. The top right and bottom right plots illustrate the evolution of horizontal and vertical divergences (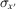 and 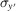), relative to their ideal values (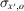 and 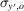). The ideal values represent beam parameters without the use of TDCs. Different colored lines correspond to the tracking results of Case #1, Case #2, Case #3 and Case #4.

To evaluate the potential impact on beam quality at locations beyond the influence of the TDCs, we conducted multi-particle tracking simulations focusing on changes in beam size and divergence. Specifically, Fig. 11 ▸ illustrates the evolution of the beam size and divergence at the midpoint of the downstream straight section adjacent to TDC2 over turns. As shown in Fig. 11 ▸, the increase in beam size and divergence individually is approximately 2%. These simulation outcomes confirm that our proposed scheme, after optimization, effectively confines the influence on beam parameters primarily to the region between the two TDCs. Consequently, beam parameters at locations beyond the TDCs are minimally affected, indicating that the beamline performance in these areas remains largely unchanged.

## 5. Conclusions and discussions

In this paper, we proposed a novel method for generating tunable double X-ray pulses in electron storage rings by utilizing a double RF system coupled with a pair of TDCs. We demonstrated how a harmonic cavity can precisely adjust the longitudinal spacing between two microbunches, enabling effective control over the time delay between X-ray pulses. Additionally, successful separation and recovery of microbunches in the vertical direction were achieved through the use of TDCs.

The peak voltage of TDC1 (*V*_TDC1_) is utilized to control the vertical displacement at the midpoint of the downstream straight section, which has a 90° vertical betatron phase advance relative to TDC1. Horizontal forces resulting from the ‘feed-down’ effects of sextupole and octupole magnets are addressed by adjusting the sextupole field strengths between the two sets of TDCs, optimized using the DE algorithm. Residual deviations in the vertical phase space are corrected by fine-tuning the settings of *V*_TDC2_ and ϕ_TDC2_.

As a result, our method allows the generation of two X-ray pulses with adjustable transverse separation and time delay, offering storage rings a novel operational scheme for scientific experiments. This capability could be further explored to broaden its application in various experimental settings.

In synchrotron light sources, adjustments to the insertion device gaps for different beamlines can alter the synchrotron radiation energy loss per turn, impacting longitudinal beam dynamics. This, in turn, changes the synchronous phase of the double RF system, affecting the longitudinal separation and arrival time of the microbunches. Consequently, these changes can influence the phases experienced in the TDCs, impacting transverse separation and the effectiveness of TDC cancelation.

To mitigate these potential perturbations, we propose several strategies: utilizing a wiggler to counteract variations in synchrotron radiation energy loss, implementing operational schemes that maintain fixed insertion device gaps during specific periods, and designing a more robust low-level RF system to ensure the stability of RF parameters. However, detailed studies are deferred to future work.

Additionally, it is important to note that the longitudinal distributions depicted in Fig. 6 ▸ were simulated in the ‘zero-current limit’, meaning that longitudinal impedance was not included. The primary reason for this approach was to focus on introducing our proposal and its foundational concepts. Recognizing that impedance effects could lead to bunch lengthening and potential-well distortion, which may reduce resolution, we plan to evaluate these effects quantitatively in future work. We intend to incorporate synchrotron radiation effects and expand our analysis to include impedance, collective effects and other factors to refine and optimize our proposal.

We are committed to thoroughly investigating these challenges and will report our findings in future work, with the goal of fully understanding and mitigating these effects.

## Figures and Tables

**Table 1:** Main parameters of the ‘toy’ lattice

Parameter	Symbol	Units	Value
Circumference	*C*_ring_	m	1360.4
Beam energy	*E*_0_	GeV	6
Radiation energy loss per turn	*U*_0_	MeV	2.887
Momentum compaction factor	α_c_	–	1.56 × 10^−5^
Energy spread	σ_δ_	–	1.061 × 10^−3^
Horizontal tune	ν_*x*_	–	114.14
Vertical tune	ν_*y*_	–	106.23
Main RF frequency	*f*_MC_	MHz	166.6
Harmonic number of main cavity	*h*_1_	–	756
Harmonic number of HHC	*h*_2_	–	2268
Horizontal damping time	τ_*x*_	ms	10.2
Vertical damping time	τ_*y*_	ms	18.9
Longitudinal damping time	τ_δ_	ms	16.5

**Table 2:** Optimized parameters for TDCs and sextupole configurations in Cases #1 to #4 Included are the voltage, phase and frequency settings for the TDCs, as well as the new sextupole strength adjustments between the two TDCs. The optimization aimed to minimize horizontal phase space displacement at the exit of TDC2 while maintaining the first-order chromaticity.

Parameters	Case #1	Case #2	Case #3	Case #4
*V*_MC_ (MV)	3.6439	3.6439	3.6439	3.6439
*V*_HC_ (MV)	0.6993	0.7288	0.7583	0.7922
ϕ_1s_ (rad)	2.0347	2.0300	2.0260	2.0217
ϕ_2s_ (rad)	5.7225	5.7354	5.7491	5.7643
Longitudinal separation (ps)	480.989	621.045	727.874	826.943
*V*_TDC1_ (MV)	0.7000	0.7000	0.7000	0.7000
ϕ_TDC1_ (°)	89.6732	89.4672	89.2394	88.9938
*V*_TDC2_ (MV)	0.7017	0.7013	0.7021	0.7016
ϕ_TDC2_ (°)	89.4293	88.0921	91.1875	90.8119
*h*_TDC_	4	4	4	4
*K*_2,*R*03*SD*1_ (m^−3^)	−200.71	−197.43	−199.06	−198.79
*K*_2, *R*03*SF*1_ (m^−3^)	190.40	186.13	187.36	186.92
*K*_2, *R*03*SD*2_ (m^−3^)	−11.46	−11.46	−11.46	−11.46
*K*_2, *R*03*SD*3_ (m^−3^)	−170.27	−178.21	−172.47	−173.29
*K*_2, *R*03*SF*2_ (m^−3^)	235.60	237.77	241.03	241.92
*K*_2, *R*03*SD*4_ (m^−3^)	−127.05	−127.47	−126.72	−126.63
*K*_2, *R*04*SD*1_ (m^−3^)	−223.68	−221.36	−223.61	−223.96
*K*_2, *R*04*SF*1_ (m^−3^)	110.55	119.82	110.10	109.77
*K*_2, *R*04*SD*2_ (m^−3^)	−75.02	−76.16	−75.02	−75.02
*K*_2, *R*04*SD*3_ (m^−3^)	−236.03	−249.20	−239.63	−239.46
*K*_2, *R*04*SF*2_ (m^−3^)	154.73	149.40	153.29	153.21
*K*_2, *R*04*SD*4_ (m^−3^]	−197.24	−192.36	−196.60	−196.29

**Table 3:** Parameters used in *SPECTRA* simulations

Parameter	Symbol	Value and units
Single bunch charge	*Q*	1.0 nC
Number of bunches	*n*_b_	648
Total beam current	*I*_b_	142.8 mA
Beam energy	*E*	6 GeV
Period length of the ID	λ_u_	19 mm
Peak *B* field of the ID	*B*_u,peak_	1.35 T
*K* value of the ID	*K*_u_	2.39501
Length of the ID	*L*_u_	2 m

## References

[bb1] Borland, M. (2000). *ELEGANT: A Flexible SDDS-Compliant Code for Accelerator Simulation*. Technical Report LS-287. Advanced Photon Source, Argonne National Laboratory, Lemont, Illinois, USA.

[bb2] Borland, M. (2005). *Phys. Rev. ST Accel. Beams* **8**, 074001.

[bb3] Borland, M., Decker, G., Emery, L., Sajaev, V., Sun, Y. & Xiao, A. (2014). *J. Synchrotron Rad.* **21**, 912–936. 10.1107/S1600577514015203 25177982

[bb4] Chao, A. W. (1993). *Physics of Collective Beam Instabilities in High Energy Accelerators.* Wiley-Interscience.

[bb5] Di Mitri, S., Barletta, W., Bianco, A., Cudin, I., Diviacco, B., Raimondi, L., Spampinati, S., Spezzani, C. & Masciovecchio, C. (2019). *J. Synchrotron Rad.* **26**, 1523–1538. 10.1107/S1600577519009901 31490140

[bb6] Emery, L., Harkay, K. & Sajaev, V. (2009). *Proceedings of the 23rd Particle Accelerator Conference (PAC09)*, 4–8 May 2009, Vancouver, BC, Canada, pp. 1084–1086.

[bb7] Eriksson, M., van der Veen, J. F. & Quitmann, C. (2014). *J. Synchrotron Rad.* **21**, 837–842. 10.1107/S1600577514019286 25177975

[bb8] Feoktistov, V. (2006). *Differential Evolution.* New York: Springer.

[bb9] Hettel, R. (2014). *J. Synchrotron Rad.* **21**, 843–855. 10.1107/S1600577514011515 25177976

[bb10] Holldack, K., Schüßler-Langeheine, C., Pontius, N., Kachel, T., Baumgärtel, P., Windsor, Y. W., Zahn, D., Goslawski, P., Koopmans, M. & Ries, M. (2022). *Sci. Rep.* **12**, 14876. 10.1038/s41598-022-19100-z PMC9437009 36050415

[bb11] Huang, X. (2016). *Phys. Rev. Accel. Beams* **19**, 024001.

[bb12] Huang, X., Hettel, B., Rabedeau, T., Safranek, J., Sebek, J., Tian, K., Wootton, K. P. & Zholents, A. (2019). *Phys. Rev. Accel. Beams* **22**, 090703.

[bb13] Huang, X., Safranek, J. & Zholents, A. (2023). *Phys. Rev. Accel. Beams* **26**, 120701.

[bb14] Jiao, Y., Xu, G., Cui, X.-H., Duan, Z., Guo, Y.-Y., He, P., Ji, D.-H., Li, J.-Y., Li, X.-Y., Meng, C., Peng, Y.-M., Tian, S.-K., Wang, J.-Q., Wang, N., Wei, Y.-Y., Xu, H.-S., Yan, F., Yu, C.-H., Zhao, Y.-L. & Qin, Q. (2018). *J. Synchrotron Rad.* **25**, 1611–1618. 10.1107/S1600577518012110 PMC6225742 30407168

[bb15] Lee, S. (2018). *Accelerator Physics*, 4th ed. World Scientific.

[bb16] Liu, L., Milas, N., Mukai, A. H. C., Resende, X. R. & de Sá, F. H. (2014). *J. Synchrotron Rad.* **21**, 904–911. 10.1107/S1600577514011928 25177981

[bb17] Martin, I. P. S., Rehm, G., Thomas, C. & Bartolini, R. (2011). *Phys. Rev. ST Accel. Beams* **14**, 040705.

[bb18] Pfingstner, J., Artoos, K., Charrondiere, C., Janssens, S., Patecki, M., Renier, Y., Schulte, D., Tomás, R., Jeremie, A., Kubo, K., Kuroda, S., Naito, T., Okugi, T., Tauchi, T. & Terunuma, N. (2014). *Phys. Rev. ST Accel. Beams* **17**, 122801.

[bb19] Raimondi, P., Benabderrahmane, C., Berkvens, P., Biasci, J. C., Borowiec, P., Bouteille, J.-F., Brochard, T., Brookes, N. B., Carmignani, N., Carver, L. R., Chaize, J.-M., Chavanne, J., Checchia, S., Chushkin, Y., Cianciosi, F., Di Michiel, M., Dimper, R., D’Elia, A., Einfeld, D., Ewald, F., Farvacque, L., Goirand, L., Hardy, L., Jacob, J., Jolly, L., Krisch, M., Le Bec, G., Leconte, I., Liuzzo, S. M., Maccarrone, C., Marchial, T., Martin, D., Mezouar, M., Nevo, C., Perron, T., Plouviez, E., Reichert, H., Renaud, P., Revol, J.-L., Roche, B., Scheidt, K.-B., Serriere, V., Sette, F., Susini, J., Torino, L., Versteegen, R., White, S. & Zontone, F. (2023). *Commun. Phys.* **6**, 82. 10.1038/s42005-023-01195-z PMC10124696 37124119

[bb20] Ries, M., Feikes, J., Goetsch, T., Goslawski, P., Li, J., Ruprecht, M., Schälicke, A. & Wüstefeld, G. (2015). *Proceedings of the Sixth International Particle Accelerator Conference (IPAC2015)*, 3–8 May 2015, Richmond, VA, USA, pp. 138–140.

[bb21] Schoenlein, R. W., Chattopadhyay, S., Chong, H. H. W., Glover, T. E., Heimann, P. A., Shank, C. V., Zholents, A. A. & Zolotorev, M. S. (2000). *Science* **287**, 2237–2240. 10.1126/science.287.5461.2237 10731140

[bb22] Streun, A., Garvey, T., Rivkin, L., Schlott, V., Schmidt, T., Willmott, P. & Wrulich, A. (2018). *J. Synchrotron Rad.* **25**, 631–641. 10.1107/S1600577518002722 PMC5929351 29714174

[bb23] Sun, C., Portmann, G., Hertlein, M., Kirz, J. & Robin, D. S. (2012). *Phys. Rev. Lett.* **109**, 264801. 10.1103/PhysRevLett.109.264801 23368570

[bb24] Tanaka, T. (2021). *J. Synchrotron Rad.* **28**, 1267–1272. 10.1107/S1600577521004100 34212893

[bb25] Tanaka, T. & Kitamura, H. (2001). *J. Synchrotron Rad.* **8**, 1221–1228. 10.1107/s090904950101425x 11679776

[bb26] Tavares, P. F., Leemann, S. C., Sjöström, M. & Andersson, Å. (2014). *J. Synchrotron Rad.* **21**, 862–877. 10.1107/S1600577514011503 PMC4181638 25177978

[bb27] Tordeux, M. A., Barros, J., Bence, A., Brunelle, P., Hubert, N., Labat, M., Nadji, A., Nadolski, L., Lebasque, P., Pollina, J. P. & Evian, C. (2012). *Proceedings of the Third International Particle Accelerator Conference (IPAC2012)*, 20–25 May 2012, New Orleans, Louisiana, USA, pp. 1608–1610.

[bb28] Venturini, M. (2018). *Phys. Rev. Accel. Beams* **21**, 114404.

[bb29] Wulff, M., Schotte, F., Naylor, G., Bourgeois, D., Moffat, K. & Mourou, G. (1997). *Nucl. Instrum. Methods Phys. Res. A* **398**, 69–84.

[bb30] Xu, H., Cui, X., Duan, Z., Guo, Y., Huang, X., Ji, D., Ji, H., Jiao, Y., Li, N., Li, X., Lu, X., Meng, C., Peng, Y., Tian, S., Wang, N., Wei, Y., Zhao, Y., Bao, W., Lin, S., Qin, L., Su, M., Zeng, F., Zhao, Z., Cao, J., Dong, Y., He, P., Kang, W., Li, J., Li, J., Pan, W., Qu, H., Wang, J., Xu, G. & Zhang, J. (2025). *Radiat. Detect. Technol. Methods* **9**, 70–81.

[bb31] Xu, H., Meng, C., Peng, Y., Tian, S., Wang, N., Cui, X., Du, C., Duan, Z., Guo, Y., He, P., Huang, X., Ji, D., Ji, H., Jiao, Y., Li, J., Li, N., Li, X., Lu, X., Liang, P., Pan, W., Qu, H., Wang, B., Wang, J., Wei, Y., Wan, J., Xu, G., Yan, F., Yu, C., Yue, S., Zhang, X. & Zhao, Y. (2023). *Radiat. Detect. Technol. Methods* **7**, 279–287.

[bb32] Xu, H.-S., Lin, C.-T., Wang, N., Xu, J.-Y. & Zhang, Y. (2024). *Nucl Sci Tech* **35**, 103.

[bb33] Xu, H.-S., Xu, J.-Y. & Wang, N. (2021). *Nucl Sci Tech* **32**, 89.

[bb34] Zhao, Z. (2018). *Synchrotron Light Sources* ch. 1, pp. 1–33. John Wiley & Sons, Ltd.

[bb36] Zholents, A. A. & Zolotorev, M. S. (1996). *Phys. Rev. Lett.* **76**, 912–915. 10.1103/PhysRevLett.76.912 10061583

[bb35] Zholents, A., Heimann, P., Zolotorev, M. & Byrd, J. (1999). *Nucl. Instrum. Methods Phys. Res. A* **425**, 385–389.

